# Recruiting children into cancer trials–role of the United Kingdom Children's Cancer Study Group (UKCCSG)

**DOI:** 10.1038/sj.bjc.6600990

**Published:** 2003-05-27

**Authors:** S Ablett, C R Pinkerton

**Affiliations:** 1 UKCCSG, 3^rd^ Floor Hearts of Oak House, 9 Princess Road West, Leicester LE1 6TH, UK; 2 The Royal Marsden Hospital, Downs Road, Sutton, Surrey SM2 5PT, UK

**Keywords:** childhood cancer; clinical trials; patient information

## Abstract

The UK Children's Cancer Study Group (UKCCSG), established in 1977, provides a highly organised structure for both service provision and research, and represents the model to which the adult cancer community is currently aspiring. Since childhood cancer is so rare, it is both essential and feasible for the vast majority of children to be referred into the network of specialist centres, and also for the maximum number of children to be recruited into national and international clinical trials. Over the last 30–40 years there have been major advances in treatment, such that now approximately 70% of children diagnosed with cancer will be cured of their disease. The conduct of clinical trials in this patient population does, however, raise a number of specific issues and these are discussed in the paper.

Childhood cancer is rare. There are approximately 1500 newly diagnosed cases each year in the UK, up to age 15 years. Over the last 30–40 years there have been major advances in treatment, and now approximately 70% of children diagnosed with cancer will be cured of their disease. When this small overall number of cases is broken down by tumour type and prognostic factors (such as age or stage of disease), the planning of clinical trials can become very difficult. Despite this, 70% of all children are currently on such trials, which are coordinated either by the UK Children's Cancer Study Group (UKCCSg) (solid tumours) or the Medical Research Council (leukaemia) (see Table 1

**Table 1:** Percentage of UKCCSG patients entered in national and international trials. Analysis by diagnostic group

**Diagnostic group**	**Years**	**Trials**	**% entered**
Acute lymphoblastic	1977–79	UKALL V–VII^a^	61
leukaemia	1980–84	UKALL VIII^a^	61
	1985–90	UKALL X^b^	74
	1991–96	UKALL XI and Infant ALL^b^	84
	1997–99	ALL 97 and Infant ALL^b^	85
			
Acute nonlymphocytic	1977–79	UKAML	43
leukaemia	1980–82	AML 8	46
	1983–87	Joint AML, AML 9	39
	1988–94	AML 10	64
	1995–99	AML 12	76
			
			
Hodgkin's disease	1982–91	8201	76
	1992–99	9201	79
			
			
NHL	1977–84	7701	54
	1985–89	8501–8503	65
	1990–95	9001–9004	81
	1996–99	9503, 9601–9603	66
			
			
PNET	1979–82	7801	44
	1985–88	SIOP	31
	1992–99	PNET–3 and Infant	59
			
			
Neuroblastoma	1982–89	ENSG Survey	83
	1990–99	ENSG 5–9, LNESG	77
			
			
Wilms' tumour	1980–85	8001	89
(incl. rhabdoid and BMRTC)	1986–91	8601	92
	1992–99	9101	95
			
			
Liver tumour	1990–93	SIOPEL–1	88
	1996–99	SIOPEL–2,3	84
			
Osteosarcoma	1983–85	EORTC 83	43
	1986–92	EORTC 86	49
	1993–99	EORTC 93	51
			
Ewing's sarcoma	1979–85	7802	61
	1986–92	8701	75
	1993–99	EICESS	78
			
Rhabdomyosarcoma	1985–88	IRS–3	21
	1989–94	MMT–89	76
	1995–99	MMT–95	79
			
All other soft tissue	1989–94	MMT–89	41
sarcoma	1995–99	MMT–95	52
			
Malignant non–CNS	1979–88	7901	70
germ–cell	1989–99	8901	88
			
CNS germ–cell	1989–95	8901	47

a Including NHL trials for T-ALL.

b Including NHL trials for B-ALL.

).

## TRIAL ACTIVITY

**Figure 1:**
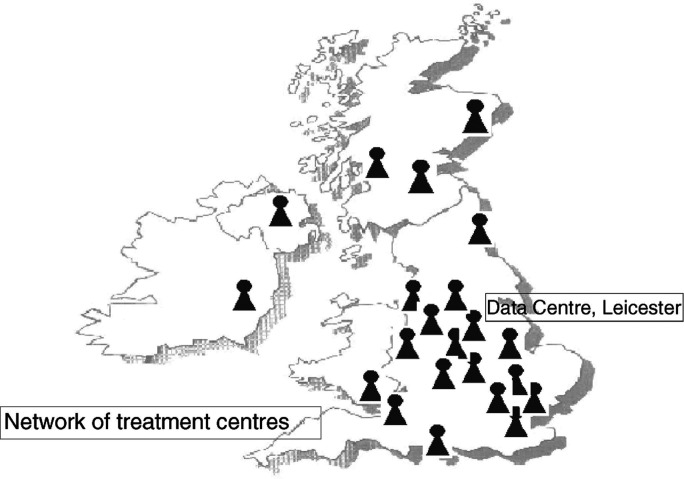
Network of UKCCSG treatment centres.

Since 1977 there has been a close-knit network of specialist treatment centres. There are now 22 of these in the British Isles and Ireland. These are all within NHS hospitals and are designated ‘UKCCSG centres’. All are recognised as centres of excellence for the treatment of children with cancer. This highly organised structure for care and research is a model to which the adult cancer community is currently aspiring through both the Calman reshaping and the National Cancer Research Network (NCRN). All of the activities of the UKCCSG are coordinated through the Data Centre in Leicester. Since childhood cancer is so rare, it is both essential and feasible for the vast majority of children with cancer to be referred to these specialist centres. Currently, around 90% of all children with cancer are treated by clinicians who are individual members of the UKCCSG. This percentage has increased steadily since the formation of the Group in 1977. At that stage, with fewer centres in place, only about 43% of children were treated in specialist centres (see Figure 1

).

The research and clinical trials activity of the Group is organised through the national structure and networks. There is a comprehensive portfolio of trials, phases I, II and III, all conducted on a multicentre basis. At any time, there will be about 30 trials open to recruitment, 10 closed to recruitment but still on active follow-up, and around 20 in advanced or early stages of planning. The last decade has seen a major increase in international collaboration. In some cases, this will mean that the UKCCSG takes a leading role in coordinating the trial, perhaps on behalf of SIOP (International Paediatric Oncology Society). In others, the collaboration may take the form of a partnership with another national group or organisation, such as SFOP (French Paediatric Oncology Society). Arrangements for overall trial coordination will vary from trial to trial. Only by combining forces to run international trials it is possible to conduct randomised trials in an acceptable time-frame, and with sufficient power to answer the study question.

Largely because of small patient numbers there is a limit to the number of early drug trials that can be run at any one time. To facilitate those trials, within the 22 UKCCSG centres there are 12 dedicated ‘phase I centres’. These must fulfil certain criteria to be able to participate in phase I trials (i.e. a committed clinician, a research nurse in post, and ability to comply with the urgent reporting and Case Report Form completion). Problems of recruitment to these early drug trials led to a decision in 1996 by the UKCCSG New Agents Group to enter into discussions with colleagues from the French Pharmacology Group. A very successful alliance has resulted from those early discussions, leading to joint phase II and I studies. The infrastructure in place within the UKCCSG Data Centre has led to it taking the main coordinating role for these studies. The alliance has recently been extended to include colleagues in Germany and The Netherlands with the creation of a European New Agents Group (EURONAG). For company-led or-supported phase I/II trials, the issues concerning recruitment are likely to be different. Financial constraints will influence the number of centres to be initiated and opened. Ensuring that every eligible patient is considered for entry on trial is crucial, so the use of screening logs has increased and is now routine in phase I trials. In this way, it is possible to monitor the reasons why some patients are not going into the trial. This may be due to lack of ethics approval, patients not fulfilling completely the eligibility criteria, or consent not being given. If recruitment is particularly slow, it may be necessary to re-examine the eligibility and exclusion criteria.

## ORGANISATIONAL STRUCTURE

As the UKCCSG has grown in size and scope of its activities, there has been continuous refinement of the organisational structure essential to its efficient management and coordination. The Executive Committee has overall responsibility for the activities of the Group, supported by the only permanent member – the Executive Director of the Data Centre. Members of the Executive, including the three Officers (Chairman, Treasurer and Secretary) are elected from among the membership and serve a period of 3 years.

Day-to-day development of clinical trial protocols and other trial-related activity is through the various working groups, representing all the tumour types and disciplines. Working Group Chairs serve for a period of 5 years and membership of the groups is regularly reviewed to maintain a balance of experience and new blood.

Central to the functioning of the UKCCSG is the Data Centre, based in the University of Leicester. Through here all the administration of the Group, as well as the national Register of Childhood Cancer, all the clinical trials, and the range of other activities, are coordinated.

**Figure 2:**
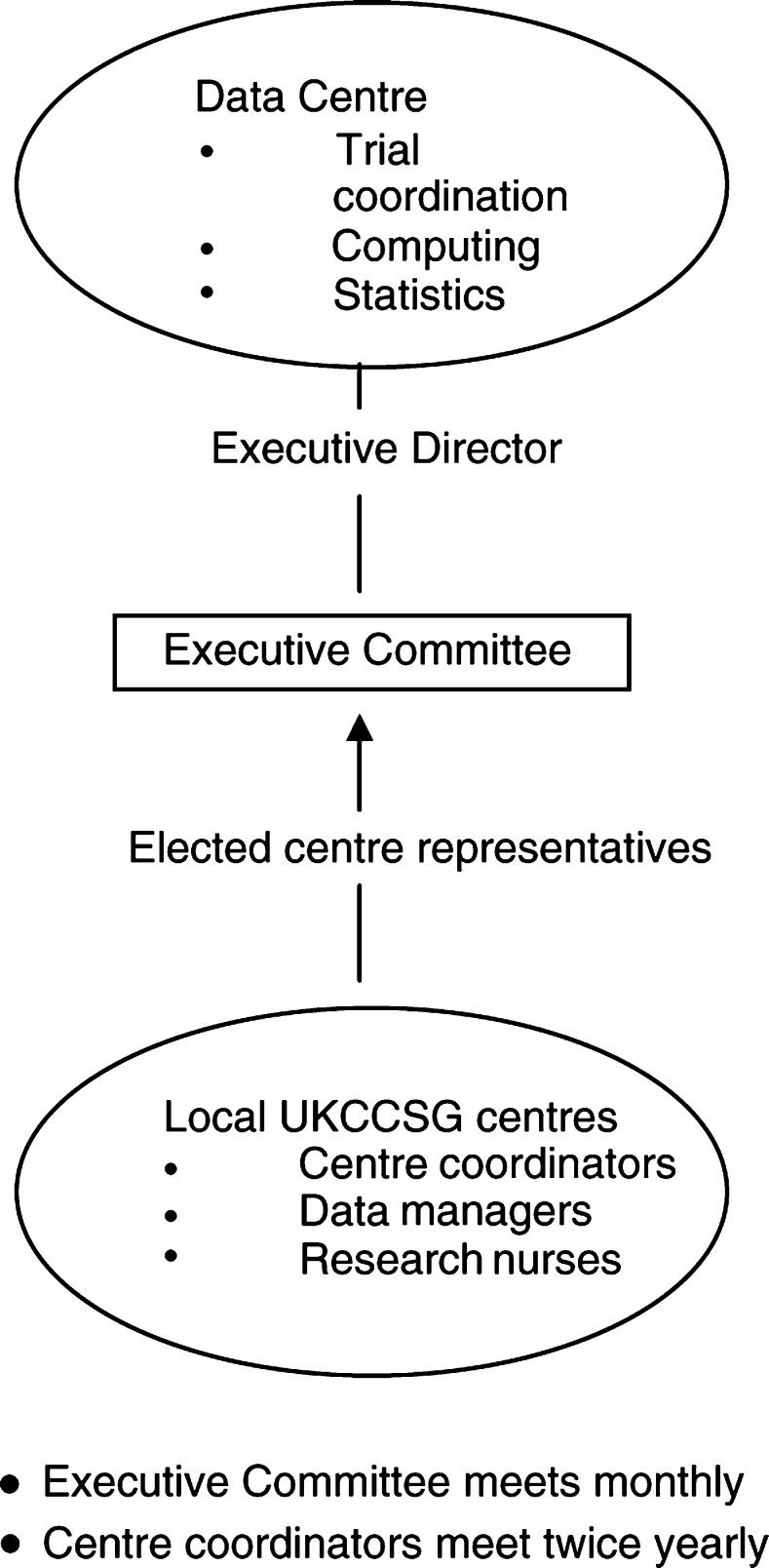
Organisational structure.

Within each UKCCSG centre, there is a named clinician who functions as ‘centre coordinator’ and whose main role is to act as a conduit for communication both to and from colleagues in the centre. Since 1990, data managers funded by the Cancer Research Campaign (now Cancer Research UK) have been employed in centres to facilitate collection of both trial and registry data. In recent years, there has been a considerable increase in the number of research nurses being appointed in the centres. Mainly financed by departmental research funds, these nurses have developed a very varied and essential role across all the trial activity of the UKCCSG (see Figure 2

).

## TRIAL DEVELOPMENT AND EXECUTION

**Figure 3:**
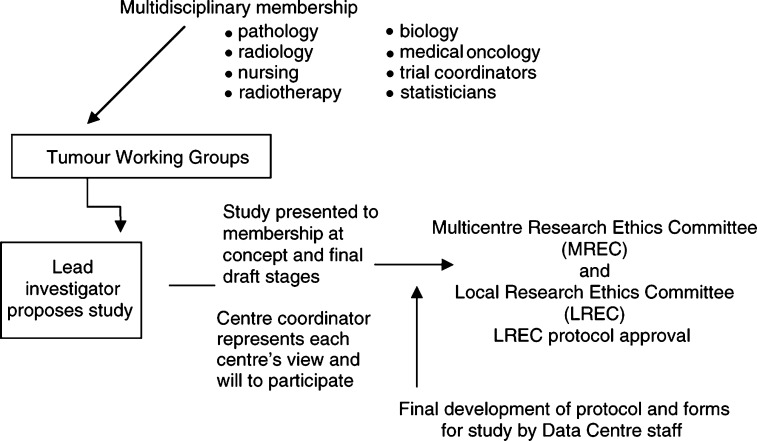
Trial development and approval.

New studies are developed through the tumour working groups including all international collaborative studies (Figure 3

).

For all trials there is a two-tier protocol approval process, which means that a protocol is first considered at one of the twice yearly national meetings as a concept and if approved, it is fully developed and submitted for final approval at the next meeting 6 months later. At both the concept and final approval stage, all UKCCSG members have the opportunity to discuss and make comment through their centre coordinator For industry trials, particularly where registration is an issue, this time-frame for approval is not always acceptable. It was, therefore, agreed that phase I trials may fast-track through the system so that once approved by the New Agents Group, they only have to come for discussion once with the national groups, thereby cutting down quite significantly on the protocol development and start up time for new phase I trials (see Figure 3).

The process of obtaining ethical approval can represent a source of potential, and sometimes significant, delay in study start up (Pinkerton *et al* (2002) *Eur J Cancer* **38**: 1051–1058). Bearing in mind that all UKCCSG trials, including phase I, will be multicentre, there is a need to obtain both national and local ethics committee approval and that will take, at best, several months before all centres are able to start recruiting patients. In the meantime, eligible trial patients may be missed.

For the UKCCSG, an average of six to eight protocols a year are opened. In the past, the principal investigator, a busy clinician, not necessarily experienced in preparing such applications, submitted the application to their nearest Multi-Centre Research Ethics Committee (MREC). It became clear that protocols were often given final approval by UKCCSG, but there was a long delay in submission to, and then approval by, MREC. Moreover, protocols were being submitted to the MREC in the region where the principal investigator was based, so that in the course of 1 year, there might have been one protocol considered at almost every MREC in the country. This was inefficient, from both the UKCCSG's and the Ethics Committee's perspective. Following discussions with Central Office for Research Ethics Committees (COREC), it was agreed that the national group could submit all protocols centrally, and to the same MREC. The applications are now prepared by the Executive Director in the Data Centre and submitted to one MREC. This new system is working well and start up times for studies are already much reduced.

Once the trial is open, data quality and trial form return rate are reviewed on an ongoing basis by staff in the Data Centre (trial coordinator and statistician assigned to a particular trial). Further clinical checking, and particularly adherence to protocol, is carried out by the study (clinical) coordinator during regular visits to the Data Centre. A facility to check and raise queries on-line will soon be available with the introduction of the first trials to be run via remote data entry. Currently, source data verification is carried out by site monitoring for phase I trials only.

**Figure 4:**
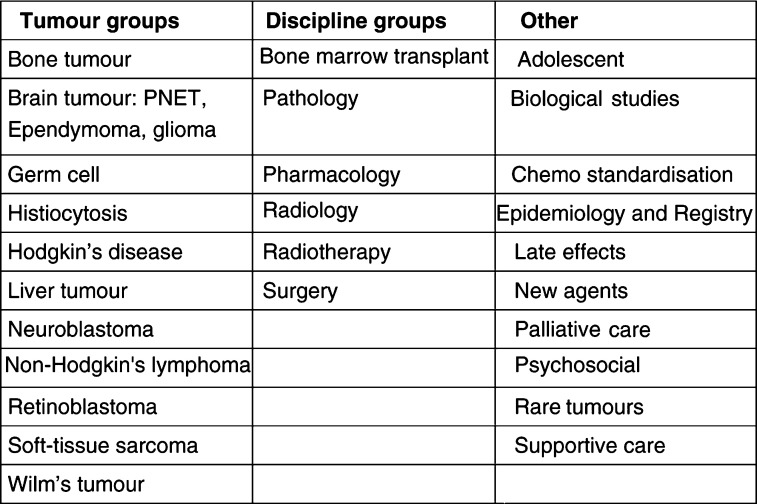
List of UKCCSG Working Groups.>

The study coordinator(s), or lead investigator(s) for any particular study also act as a main point of contact, either by telephone or e-mail, for discussion about difficult cases. Where there is a query related to imaging or pathology, for instance, then the lead radiologist or pathologist on the working group is consulted. Issues around chemotherapy may lead to contact with the pharmacist on the group, or reference to the UKCCSG Chemotherapy Standardisation Group (see Figure 4

).

## INFORMING THE PARTICIPANTS

Parent/Patient Information Sheets are now a crucial part of any treatment protocol, and are an area on which the ethics committees place a great deal of importance. Providing clear and detailed information can help in reassuring both parents and children about the safety of the trial. Provision of such information to parents and patients is a major issue. Under the new (1998) Data Protection Act, it is a legal requirement in order that consent can be considered to be truly informed. To ensure that the information provided about the safety and nature of the trial is clear, a first requirement is that the language should be appropriate, whether the information is being given orally or in writing, and if necessary translated into another language. In practice, the information is given both orally and in writing, and on a number of separate occasions. Timing of conveying any information to families and patients can be critical. Of particular difficulty may be the study design requiring day 1 randomisation when the clinician has just met the family, has to explain that their child has cancer, invite participation into a clinical trial, and then raise the question of randomisation. Much of the terminology used will be completely unfamiliar at this stage. Care must be taken to avoid information overload, and to ensure that the situation is handled in the most sympathetic and effective way possible.

A template Patient Information Sheet has been produced for use with all MREC submissions and plans to develop a similar template for children are underway. The guidelines for preparation of Patient Information Sheets for children in the UK include the need for age-specific Patient Information Sheets. Three of these are now required: one for children aged 14+ years (the wording of which will be almost identical to that for the parents); one for children aged 8–14 years (with appropriate wording); and a third for children under the age of 8 years, with the expectation that this will be read to the child by the parent or guardian. The latter may include pictures, if relevant and appropriate. Devising these for some of the particularly complex protocols is proving especially challenging. Where eligibility includes young adults, who are legally able to consent for themselves (over 18 years in England, Wales and Northern Ireland, and over 16 years in Scotland), further information sheets/consent forms are required.

Both the wording of the information, and the way it is communicated to the parents or patients, may have an impact on whether or not they decide to participate in a trial. Sufficient time must be allowed for this procedure in the centre. The arrangements in the centres will vary, but it is likely to be both the clinician and the research nurse who are involved in this process.

For the phase I and II studies, the role of the local research nurse is crucial. The relationship with the parents and children is one that is built on trust, and that may have been developed over a considerable time. It is important that there is honesty about the risks or hazards that may be involved with any trial. The parent or child needs time to absorb the information given, and to ask questions, either at the time, or later, and clear contact details must be provided. They need to fully understand that participation is voluntary, and that they are free to withdraw at any time without jeopardising either their future treatment or the relationship with the doctor or team. Further reassurance about safety in the trial can be given by reference to the very close monitoring that will take place, particularly of any serious toxicities reported, and that if necessary the trial could be stopped early. Parents and children need to know that they will be kept informed of any significant developments within the trial that may affect safety.

One of the main difficulties about providing information in this setting, and ensuring that it is fully absorbed, is that it is often being delivered at a time of maximum stress for the families. To try and ensure that information is readily available at any time, the UKCCSG now produces a quarterly magazine for families of children and young people with cancer. Produced now in collaboration with the National Alliance of Childhood Cancer Parent Organisations (NACCPO), *Con*tact represents a very positive example of professionals and parents working together. One of the primary aims of the magazine is the provision of good and reliable information, on a whole range of topics. The content takes account of the fact that the readers will include the newly diagnosed, those at the palliative care stage, the recently or the not so recently bereaved parents, extended family and friends, as well as health-care professionals and the general public. The articles are written for the lay person, and with 9000 copies per issue being distributed and available in each centre, wide national coverage is ensured. *Con*tact is now also available on the web (www.ukccsg.org/contact_magazine.htm). This magazine has enabled the UKCCSG to flag up the importance of research and specific trial-related matters, such as the different phases of clinical trials, and the aims and particular issues surrounding each. Reader feedback has confirmed that these are the sort of articles that readers find interesting and helpful. The hope is that by providing clear and detailed information, and taking time to discuss it thoroughly, parents or children will be enthusiastic to participate in clinical trials.

## FUNDING OF ACTIVITY

The UKCCSG receives no permanent funding. Running costs of all of the activities conducted under the umbrella of the UKCCSG now exceed £1.25 million a year. Of this, grant income makes up about three-quarters, with the remainder from charitable donations, pharmaceutical company-sponsored trials, and a levy paid by each of the centres in relation to research activity undertaken in each centre. The main source of grant income has for many years been The Cancer Research Campaign (now Cancer Research UK) both in the form of peer reviewed core funding and specific project grants. Grants are also obtained from a number of smaller charities.

## CONCLUSION

Overall referral into specialist centres for children with cancer, and recruitment into clinical trials is very high and exceeds considerably the targets currently being set for the NCRN for the adult cancer trials in the UK. There are, however, still tumour types (e.g. brain tumours) and age groups (e.g. adolescents), where centralised care is less well organised and trial recruitment remains low. There is also some variation between centres in terms of facilities, staffing and independent funding, which may lead to differences in recruitment to trials and biological studies in particular. There also remain issues about UK randomisation rates to certain studies in comparison with other European centres.

